# Stepped implementation-to-target: a study protocol of an adaptive trial to expand access to addiction medications

**DOI:** 10.1186/s13012-022-01239-y

**Published:** 2022-09-29

**Authors:** James H. Ford, Hannah Cheng, Michele Gassman, Harrison Fontaine, Hélène Chokron Garneau, Ryan Keith, Edward Michael, Mark P. McGovern

**Affiliations:** 1 School of Pharmacy, Social and Administrative Sciences Division, University of Wisconsin, Madison, USA; 2 Department of Psychiatry and Behavioral Sciences, Division of Public Health & Population Sciences, Center for Behavioral Health Services and Implementation Research, Stanford University School of Medicine, Palo Alto, USA; 3 Division of Behavioral Health & Recovery, Washington State Health Care Authority, Olympia, USA; 4 Department of Medicine, Division of Primary Care and Population Health, Stanford University School of Medicine, Palo Alto, USA

**Keywords:** Medications for opioid use disorder, Addiction treatment, Implementation strategies, Adaptive trial design

## Abstract

**Background:**

In response to the US opioid epidemic, significant national campaigns have been launched to expand access to `opioid use disorder (MOUD). While adoption has increased in general medical care settings, specialty addiction programs have lagged in both reach and adoption. Elevating the quality of implementation strategy, research requires more precise methods in tailoring strategies rather than a one-size-fits-all-approach, documenting participant engagement and fidelity to the delivery of the strategy, and conducting an economic analysis to inform decision making and policy. Research has yet to incorporate all three of these recommendations to address the challenges of implementing and sustaining MOUD in specialty addiction programs.

**Methods:**

This project seeks to recruit 72 specialty addiction programs in partnership with the Washington State Health Care Authority and employs a measurement-based stepped implementation-to-target approach within an adaptive trial design. Programs will be exposed to a sequence of implementation strategies of increasing intensity and cost: (1) enhanced monitoring and feedback (EMF), (2) 2-day workshop, and then, if outcome targets are not achieved, randomization to either internal facilitation or external facilitation. The study has three aims: (1) evaluate the sequential impact of implementation strategies on target outcomes, (2) examine contextual moderators and mediators of outcomes in response to the strategies, and (3) document and model costs per implementation strategy. Target outcomes are organized by the RE-AIM framework and the Addiction Care Cascade.

**Discussion:**

This implementation project includes elements of a sequential multiple assignment randomized trial (SMART) design and a criterion-based design. An innovative and efficient approach, participating programs only receive the implementation strategies they need to achieve target outcomes. Findings have the potential to inform implementation research and provide key decision-makers with evidence on how to address the opioid epidemic at a systems level.

**Trial registration:**

This trial was registered at ClinicalTrials.gov (NCT05343793) on April 25, 2022.

**Supplementary Information:**

The online version contains supplementary material available at 10.1186/s13012-022-01239-y.

Contributions to the literature

• Stagewise Implementation-To-Target–Medications for Addiction Treatment have elements of a sequential multiple assignment randomized trial (SMART) design and a criterion-based design that employs a measurement-based stepped implementation-to-target approach within an adaptive trial design to improve access to medications for opioid use disorder (MOUD).
• The trial is conducted in a real-world healthcare setting (specialty addiction programs).
• MOUD access in specialty addiction programs has lagged in both reach and adoption—making it challenging for patients with opioid use disorder who need and want medications to receive it.
• Findings could advance drug abuse treatment research by optimizing strategies to implement MOUD.

## Background

The 21st Century Cures Act allocated unprecedented funding to combat the US opioid epidemic. Its mission is to reduce opioid over-prescribing, improve access to overdose rescue medications, and increase the adoption and delivery of medications for opioid use disorder (MOUD) [1, 2]. Efforts have been primarily focused on expanding access to MOUD. The Substance Abuse and Mental Health Services Administration (SAMHSA) State Targeted Response and the State Opioid Response grants to 57 US states and territories sought to expand access to methadone, buprenorphine, and naltrexone across a variety of settings and systems [2–4].

While indications of improved access across a variety of settings exist, an ironic gap persists [5–10]. Primary care practices, emergency departments, and the criminal justice system have increasingly adopted MOUD, but traditional specialty addiction treatment programs have not progressed. Currently, only 35.5% of addiction treatment programs offer MOUD [6]. As such, only 15% of patients in specialty addiction treatment with opioid use disorder (OUD) are receiving MOUD [5, 11]. In fact, general medical practice settings are now twice as likely to offer MOUD than specialty addiction treatment programs [12–14].

Specialty addiction treatment programs are under pressure to implement MOUD but face many barriers [7, 10, 15–18]. Public health systems now support MOUD in policy and financing, but multiple contextual barriers persist, ranging from an abstinence-based philosophy that conflicts with pharmacological approaches, a lack of network connectivity with other health care organizations, and program structure and workflows based entirely on psychosocial interventions and peer recovery supports [19–21]. Although there has been progress among some specialty programs, most lag behind in their efforts to offer MOUD to their clients [11].

### Implementation research to improve MOUD access

This study builds on our prior implementation research. In specialty addiction programs, a cluster randomized controlled trial with addiction treatment organizations located in the State of Washington utilized a multi-level strategy—NIATx (Network for the Improvement of Addiction Treatment) [22–26] delivered through external facilitation (NIATx-EF) to implement integrated mental health services for persons with co-occurring psychiatric and substance use disorders [27]. Overall, the NIATx-EF implementation strategy had a significant effect on improving integrated services [28] and increasing patient access to substance use and psychotropic medication, as well as decreasing wait times from diagnosis to medication receipt [29, 30]. We also found that NIATx-EF was effective, more effective with NIATx-EF adherence, and surprisingly, that 10% of organizations achieved target implementation outcomes early on with only enhanced feedback using a standardized quality measure, Dual Diagnosis Capability in Addiction Treatment (DDCAT).

Four system-level, naturalistic cohort evaluation studies focused on implementing MOUD in public health care systems. In Vermont, a learning collaborative implementation strategy showed increased guideline adherence and reduced variation but no change in reach or adoption [31]. Two California-based studies examined how exposure to different implementation strategies impacted reach (number of patients receiving MOUD) and adoption (number of eligible providers prescribing MOUD). While both studies showed a significant change in reach and adoption, the influence of specific implementation strategies was mixed [32–34]. The final study found that access to implementation support: external facilitation, learning sessions, didactic webinars, and enhanced monitoring and feedback (EMF), resulted in significant improvement in implementation quality [35]. Although mitigated by the lack of controlled experimental designs, two major takeaways were that some practices make changes with a “soft touch” implementation strategy support—such as EMF—using standardized measures like the DDCAT and target performance measures, and offering a buffet of implementation support options is unnecessarily burdensome, inefficient, and costly.

### Multi-level implementation strategies to improve MOUD access

Implementation science continues to enhance scientific rigor by systematically examining implementation strategies as the “interventions” of implementation. More recently, experts have called for implementation strategies to be examined by (1) their discrete and combined components, (2) conceptualized mechanisms of action, (3) how they may be tailored or adapted to context or other factors, (4) how they may be delivered with fidelity, (5) how enrolled participants engage and finish, (6) how alternative strategies compare with one another on implementation outcomes, and (7) how much they cost [36–39]. Although exemplar studies featuring some of these expert recommendations for implementation strategies do exist, none to date features all, and none is in the critical field of implementing MOUD.

**Fig. 1:**
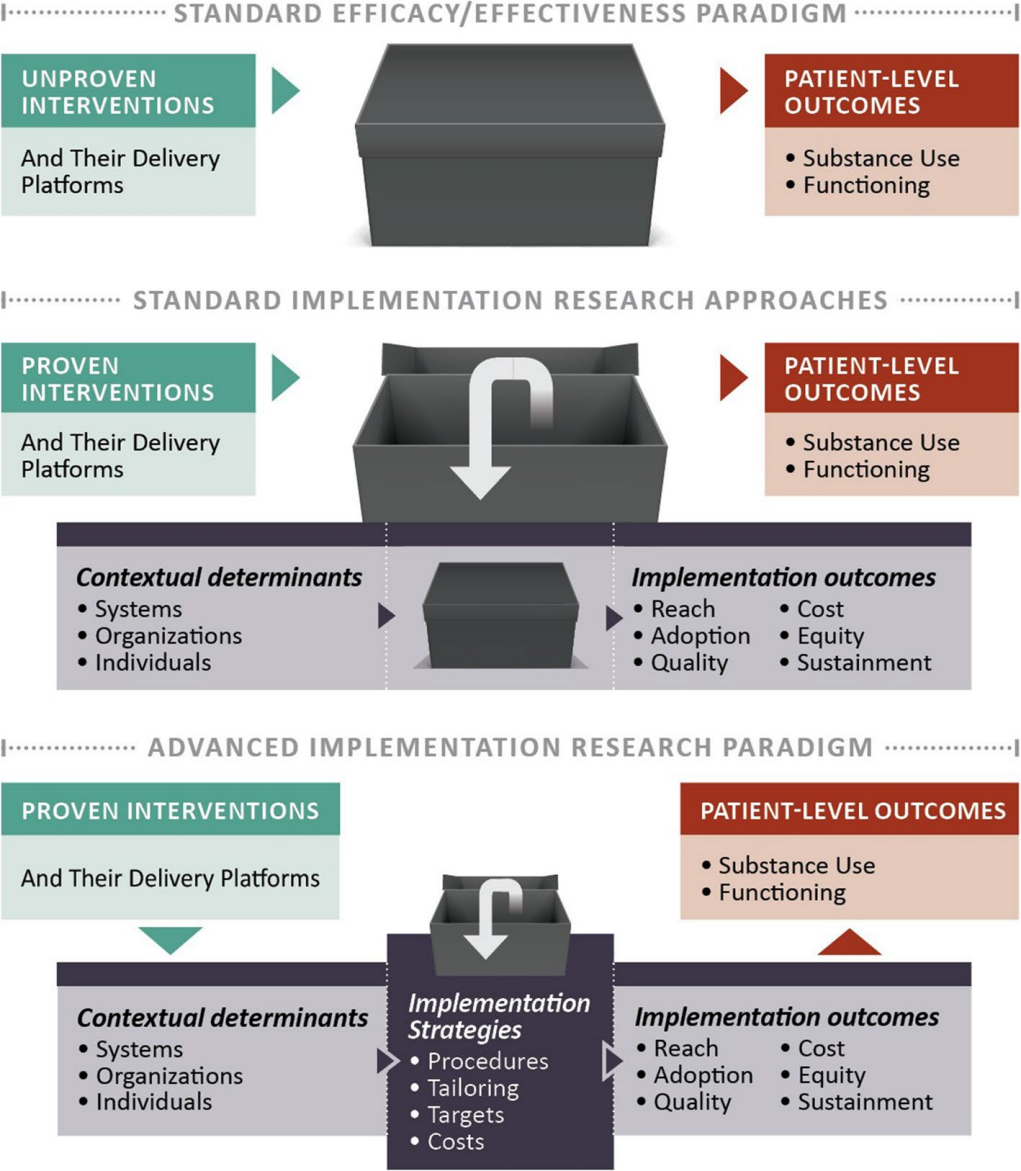
Opening the “black box” of implementation

The new paradigm for implementation research (Fig. 1) in addiction health services must extend beyond just opening the “black box” of implementation context and outcomes [33]. Specifically, the quality of implementation strategy research must examine the contents of the “black box” as shown in the final level as depicted in Fig. 1 to better understand how and why an implementation endeavor succeeds or fails. In response to expert recommendations, more precise methods are necessary to tailor implementation strategies rather than a one-size-fits-all-approach, document participant engagement and fidelity to the delivery of the strategy, and conduct economic evaluations to inform decision making and policy. Research has yet to incorporate all three of these recommendations to address the challenges of implementing and sustaining MOUD in specialty addiction programs. The Stagewise Implementation-To-Target–Medications for Addiction Treatment (SITT-MAT) study will address both the public health care and the scientific quality gaps.

## Methods

### Aims and objectives

The overarching goals of the SITT-MAT project are to equally advance implementation science and to solve a persistent public health care problem, access to addiction medications in specialty addiction treatment programs. The study aims to (1) evaluate the relative impact of a sequence of four implementation strategies on target outcome criteria within a stepped measurement approach which includes a randomized implementation trial, (2) examine contextual moderators and mediators of performance on target outcomes as a function of implementation strategy step, and (3) document the costs associated with participating in and delivering the sequence of implementation strategies and to model costs per implementation strategy to achieve target outcome criteria.

### SITT-MAT conceptual model

**Fig. 2:**
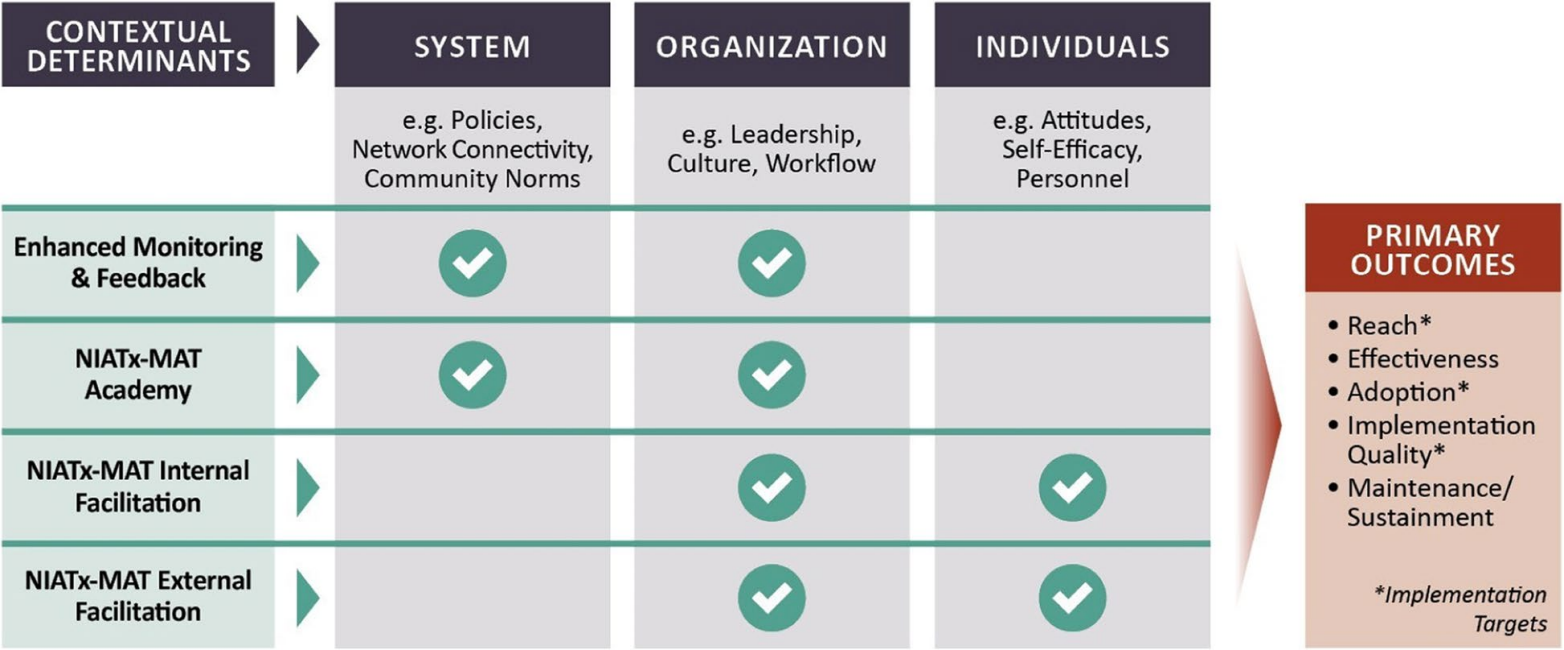
SITT-MAT conceptual model: aligning multi-level strategies with contextual determinants

Advances in the characterization of context, and the evaluation of outcomes, demonstrate methodological progress for implementation science. Our dynamic non-linear conceptual model is based upon matching implementation strategies to levels of contextual determinants (i.e., barriers or facilitators) to produce desired outcomes (Fig. 2) [38, 40, 41].

The characterization and measurement of the contextual determinants can be organized at three levels: outer (systems and community), inner (the organization or setting), and individual (providers and staff) perspectives along with perceptions of the implemented intervention are determinants of successful or unsuccessful implementation [40, 42]. These determinants can be assessed through the Consolidated Framework for Implementation Research (CFIR) which provides an inventory of barriers and facilitators (Table 1) across the three different levels [40].

**Table 1:** System, organizational, and individual contextual barriers to MOUD

**System-level factors**	System-level factors include policy, community, and financial levers. In MOUD implementation by specialty addiction programs, system barriers include myths about prohibitive policies, historical isolation from other health care providers, and norms that do not endorse MOUD
**Organization-level barriers**	Organization-level barriers include leaders who may not support MOUD, a culture of anti-MOUD as “just another crutch” and inflexible daily schedules
**Individual contextual barriers**	Individual contextual barriers are manifest in one’s own personal experience with addiction recovery, the lack of physicians or any licensed prescriber, and addiction counselors’ fears about the “professionalization” of the field

Implementation strategies might address potential barriers at each level to implement MOUD, but presently, no solid empirical bases for selection and tailoring based on contextual determinants exist [43, 44]. Instead, we were guided by four criteria in our selection of implementation strategies: (1) the evidence for each strategy, (2) the standard practice of using the strategy to address typical implementation problems at the determinant level, (3) our own research, and (4) the incremental effort and cost associated with each strategy.

### Study design

**Fig. 3:**
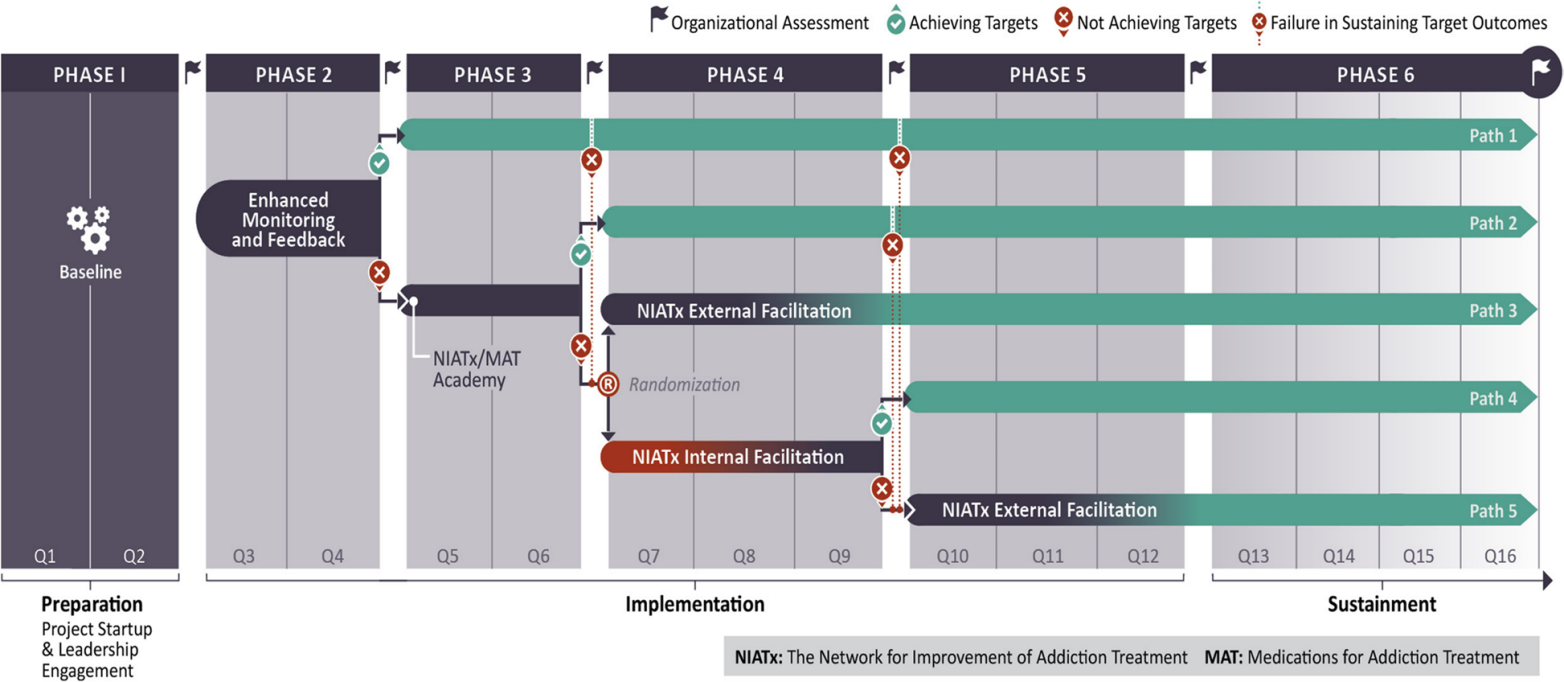
SITT-MAT adaptative implementation strategy design

We will deploy an adaptive implementation strategy design that incorporates a nonrandomized evaluation and a randomized implementation trial (Fig. 3). The stagewise implementation-to-target, stepped approach to implementation, with adaptation based on actual performance, is soundly based on our prior research and a reasonable conceptual model. The stagewise implementation design is like the idea of offering a “light touch” implementation strategy to programs that require minimal support to achieve targets, and progressing towards additional strategies that are more intense, resource-demanding, and costly, but only if needed. Four implementation strategies are staged sequentially, based on the program-level response (Table 2).

**Table 2:** SITT-MAT implementation strategies

**Strategy**	**Description**
**Enhanced monitoring and feedback (EMF)**	All participants will receive EMF initially and throughout the course of the active implementation phase of the study. EMF consists of the following: • Performance data gathered and reported by the program for reach and adoption on a quarterly basis • Reach, adoption, and effectiveness data summaries and IMAT results for each program, and, in comparison to the entire sample, will be reflected back to clinical leaders and staff members, via dashboards
**NIATx/MAT academy**	Two-day workshop: • Day 1 – Provide participating programs with rationale, clinical practice, and program integration with MOUD. Dr. McGovern and regional MOUD expert leader prescribers will present clinical aspects of MOUD, integrating MOUD into culture and workflow, and managing complex situations • Day 2 – Provide participating programs with an overview of NIATx principles and tools. Dr. Ford, who has used the NIATx academy to train over 1000 individuals, will provide participants with the critical skills (e.g., building change teams, conducting walkthroughs) and tools (e.g., process flow charts, nominal group technique) needed to implement rapid cycle Plan-Do-Study-Act (PDSA) process improvement
**NIATx-internal facilitation (NIATx-IF)**	Internal facilitators will receive training on how to provide coaching within their organization. For 9 months, they will do the following: • Support teams to harness resources toward systemic change and improvement • Employ a range of concepts and tools to provide individualized support to teams • Help teams to practice and hone their skills to optimize performance • Focus on team relationships and communications • Participate in group coaching calls involving IFs from other NIATx-IF programs and moderated by NIATx expert to discuss common change-related issues, progress, and successful tactics
**NIATx-external facilitation (NIATx-EF)**	For 9 months, organizations in the NIATx-EF study arm will work with one of 4 experienced consultant NIATx-external facilitators (EFs). The EF will do the following: • Meets with clinic staff to plan change projects, review ongoing change efforts, discuss successes, and offer guidance on planning future change projects using PDSA cycles • Makes a 1-day site visit during the 1st quarter of the implementation period • Leads monthly 1-h phone calls

#### Implementation strategies

The first implementation strategy is enhanced monitoring and feedback (EMF), which has some elements of “audit and feedback” widely used in the Veterans Health Administration [44–46]. This strategy is provided to all participating programs. The EMF incorporates the use of performance data, compiled, and reflected back to programs with normative status. It targets contextual determinants at the system and organizational level. As we found in our prior studies to improve co-occurring capacity [28, 47], we hypothesize that an EMF condition, providing dashboard feedback on reach and adoption plus a measure of implementation quality, will produce quick outcomes for some programs. We predict that this strategy will most likely work with programs with fewer barriers that do not need as much support to meet target outcomes.

The second implementation strategy is the NIATx/MAT Academy that is provided if EMF is not immediately successful. The academy is a 2-day training/workshop that will bring together members of specialty addiction programs from across the state, debunk myths about MOUD and abstinence-based philosophy, and instruct on MOUD content and NIATx implementation processes. This strategy aligns with systems and organizational determinants. Our prior research found a positive impact of learning sessions on reach and adoption, as well as implementation quality [31, 33, 35].

All programs that do not achieve or sustain target outcome criteria by the end of Phase 3 (12 months from baseline) will be randomized to either of the remaining two implementation strategies—NIATx Internal Facilitation (NIATx-IF) and NIATx External Facilitation (NIATx-EF). The NIATx-IF strategy features an internal change leader (employee) who will help other staff gain skills and efficacy to implement changes and address barriers in attitude and beliefs. Thus, determinants at the individual and organizational levels are the focus of the NIATx-IF implementation strategy (Fig. 2). NIATx-EF is an evidence-based implementation strategy that is widely used and studied [45]. NIATx-EF features an external expert or coach. The coach leverages their expertise and knowledge to guide staff in implementing changes, help employees gain confidence and skills, and address potential obstacles including stigma and attitudes [22, 48]. For the same reasons as NIATx-IF, NIATx-EF is tailored to issues at the organization and individual levels. IF and EF models have been systematically studied and compared [46, 49, 50]. Although there is evidence for both IF and EF, with better outcomes for EF, the cost for EF is likely greater than for IF [49]. Using the procedures employed in a previous implementation trial, we will balance the two groups before randomization [51]. We will then use a randomized cluster group trial design to compare NIATx-IF to NIATx-EF.

In our study design, programs that “graduate” to the sustainment/follow-up arm but then fail to maintain the criteria of success for two consecutive quarters will have two opportunities to rejoin the active implementation strategy phases (Fig. 3). The first opportunity where a program on Path 1 could re-enter is at the end of Phase 3 if they do not sustain improvements after receiving the EMF strategy. The second opportunity for a program on Path 2 to re-enter would be at the end of Phase 4 if they do not sustain improvements after receipt of the EMF and NIATx/MAT Academy implementation strategies. There are thus five possible paths a program might take in this study. Months in the active implementation stage range from 6 (Path 1) to 30 (Path 5). The sustainment period ranges from 12 months (Path 5) to 36 months (Path 1). The path followed by a program depends on when it meets (and sustains) implementation targets.

This study was reviewed by the Stanford University Institutional Review Board and approved as a minimal risk study (eProtocol # 66,398). The project is registered at ClinicalTrials.gov (NCT05343793). The Standards for Reporting Implementation Studies (StaRI) reporting standards and checklist (Additional File 1) was used [52].

### Participants

We will enroll 72 addiction treatment programs from the State of Washington. Programs that are either residential (detoxification or rehabilitation) or outpatient (intensive outpatient or outpatient) levels of care are eligible. The community addiction treatment programs in Washington are comparable to addiction treatment programs nationwide in MOUD implementation. Specifically of all addiction treatment programs in the state, 72% do not offer MOUD. Therefore, despite the potential for limiting inference outside of one system, findings should be generalizable. Opioid treatment programs (i.e., methadone clinics) are not eligible because they are already engaged in MOUD.

Near the conclusion of the project, efforts will be made to examine aggregate administrative data obtained from the State of Washington Health Care Authority, regarding MOUD activity (reach, adoption), the population of addiction treatment programs across the state. This will enable a naturalistic comparator to the volunteer effects of study participation and also the influence of historical factors (e.g., major changes in policy, financing, or public health circumstances [viral epidemics]) that might have impacted the entire state.

A qualitative approach to understanding the participating organizations’ experience with contextual factors, the sequence of strategies, and changes in the culture and practice would be of great methodological value. To achieve this goal, we will conduct key informant interviews with 10% of participating organizations, stratified to represent the range of participant characteristics, e.g., start-up vs. scale up, urban/rural, level of care (residential/inpatient; outpatient/intensive outpatient).

### Outcomes and data collection

Three categories of measures are collected in the SITT-MAT study: (1) primary implementation outcomes (Aim 1), (2) contextual determinants (Aim 2), and (3) implementation strategy participation, fidelity, and cost measures (Aim 3). Although all measures interrelate to address the study aims, each category is principally associated with an aim as specified below.

### Implementation outcomes (Aim 1)

Primary outcomes are organized by the RE-AIM taxonomy and the Addiction Care Cascade [53–55]. Reach, Effectiveness, Adoption, Implementation (quality of MOUD), and Maintenance (sustainment) are augmented by timely access and retention in care—the latter two are elements of the Addiction Care Cascade (Table 3). The Reach, Adoption, and Effectiveness outcomes have been similarly collected using the identical procedure in our recently completed study [35].

**Table 3:** Primary outcome definitions

	Aim 1	Aim 1 definition
**Aim 1 primary outcomes**	Reach	The proportion of program patients with OUD and receiving MOUD (buprenorphine, naltrexone) within the index quarter
	Adoption	The number of onsite integrated DEA x-waivered prescribers of buprenorphine or prescribers of naltrexone, who are prescribing MOUD
	Effectiveness	*Access*: The proportion of patients prescribed MOUD who start the medication within 72 h of OUD diagnosis. For patients requiring detoxification for naltrexone, *Access* will be operationalized as the proportion of patients who start the medication within 72 h of when it is safe *Retention*: The proportion of patients who are retained in continuous care for at least 6 months from the start of medication, or if in time-limited care situations (e.g., residential detoxification) for the entire treatment episode
	Implementation	Changes in the IMAT Index at the assessment points are indicated in Fig. 3
	Maintenance	To assess sustainment, the primary outcomes detailed above will be monitored quarterly, even as an organization moves into the sustainment phase. IMAT data collection for the organizations in the sustainment phase will follow the same timeline as that of organizations still engaged in the active implementation (post each strategy, and at 1-year follow-up) Together, these four outcomes (RE-AI) will reflect the sustainment of gains made during the active implementation phase as a function of (a) implementation strategies, (b) contextual determinants, and (c) participation in and fidelity to the implementation strategies

Implementation quality will be measured using the Integrating Medications for Addiction Treatment (IMAT) Index. The IMAT is a newer measure built upon the same framework and methodology as its widely adopted predecessors [26, 56, 57]. The IMAT, comprised of 45 items clustered into seven dimensions (Additional File 2), integrates MOUD guidelines, expert consensus recommendations, the OUD care cascade, and best practice information into a team-assessment benchmark measure of current and future state MOUD capability and practice [58]. Confirmatory factor analyses support the validity of the IMAT Total Score, as well as the validity of each dimension. Reliability scores for each dimension range between 0.70 and 0.95. The IMAT has been useful to systems, organizations, and teams as an objective measure of current state MOUD practice and as a blueprint for measurable practice change. Like the DDCAT, the IMAT is transparent in depicting what is needed to score a 3 or 5 for practices seeking to improve implementation quality.

Three primary outcomes-Reach, Adoption, and Implementation (IMAT) serve as implementation targets to determine the stagewise path of participating organizations. A program will meet the SITT-MAT adaptative assessment criteria if (1) greater than or equal to 50% of their patients with OUD are receiving a MOUD (Reach), (2) the program has an integrated MOUD prescriber who is employed or contracted by the program (Adoption), and (3) the IMAT Total Score is greater than or equal to 3 (Implementation). By including a measure of intervention quality (IMAT), our study will overcome the threat of implementation without fidelity being no better or even harmful [59, 60]. The Reach and Adoption primary outcome measures will be gathered at baseline and quarterly for the duration of the trial, to the end of the sustainment phase and the IMAT-SC will be assessed at baseline and at the end of each phase (Fig. 3).

#### Contextual determinants (Aim 2)

Contextual determinants are levels of the system, organization, or the individuals working within the organization that influence implementation outcomes. In addition, there are physical characteristics of the organization itself, such as size, location (urban/rural), or level of care that may influence implementation outcomes. We selected three commonly used measures associated with two major contextual determinant frameworks: Consolidated Framework for Implementation Research (CFIR) and the Exploration, Preparation, Implementation and Sustainment (EPIS) [42].

In this study, we developed the contextual determinant inventory (CDI) following the CFIR, EPIS, and Health Equity Implementation Framework [61]. The CDI’s 42 items are organized in four levels: system, program, staff, and patient. The rating scale ranges from Strongly Disagree to Strongly Agree and includes a Does not apply option. A 43rd item is an open-ended question asking about other contextual determinants that may affect implementation but were not included in the CDI. Scores can be tallied overall, for the 42 items, by individual items, or by dimension. Like the IMAT, the CDI is a team-assessment measure completed by key staff in MOUD implementation.

Based on the EPIS, the Implementation Climate Scale (ICS) includes 6 subscales and 18 items scored on a 5-point scale from 0 – not at all, to 4 – very great extent [62]. The Implementation Leadership Scale (ILS) is composed of 12 items divided into four subscales [63, 64]. The items are rated on a 5-point scale from 0 – not at all, to 4 – very great extent. The ICS and ILS are surveys completed by at least 5 staff members at each organization. Each of these measures will be collected at baseline, at the 4 implementation intervals, and at any 1-year post-implementation support.

#### Implementation strategy participation, fidelity, and costs (Aim 3)

Advanced measures of implementation strategy participation, fidelity, and cost are the key study features that increase precision and scientific rigor. We will use four measures to detail the contents of the “black box” of implementation strategies: NIATx Fidelity Scale, Stages of Implementation Completion (SIC), Costs of Implementing New Strategies (COINS), and an Extended CONSORT diagram to describe how programs stepped through the different implementation strategies.

The *NIATx Fidelity Scale* is a 19-item observational measure of adherence to the NIATx model, rating activities on a 5-point scale from *1-No evidence to 5-Extensive evidence*. Scoring reflects information obtained from IF and EF perspectives using a composite of interviews, review of walk-through results, change project forms, EF or IF notes, and sustainability plans. The research team will monitor NIATx delivery and formally assesses fidelity by utilizing an online data collection tool with input from external facilitators (e.g., coaches) after each strategy of the active NIATx-IF and NIATx-EF implementation phase. Where significant deviations are identified at the first fidelity check, corrective adjustments can be made.

The SITT-MAT Adaptative Implementation Strategy Design involves four strategies (EMF, NIATx/MAT Academy, NIATx-EF, NIATx-IF) and five possible pathways for programs that participate. Therefore, the current project will include assessments of strategy adherence across the five possible paths using the *Stages of Implementation Completion (SIC)* and the resources required using the *Cost of Implementing New strategies (COINS)*.

The SIC is an 8-stage assessment tool that is psychometrically valid and reliable [65–69]. The SIC is designed to measure and compare implementation strategies for scaling up proven interventions [51]. Stages range from engagement (Stage 1) to achievement of program delivery with competency (Stage 8) [67]. SIC data include a log of activities that operationalize the implementation process necessary to move toward successful program start-up and sustainment and their completion dates. Two scores are calculated for each SIC stage. The *Proportion* score calculates the proportion of key activities completed within a stage. The *Duration* score is calculated by the date of entry through the date of the final activity completed. The Duration score can account for activities not completed sequentially and for being in multiple stages at a given time. A third *Final Stage* score indicates the final stage achieved in the implementation process (Stages 1–8). The SIC has been adapted or customized for multiple evidence-based practices (EBPs) [70–75] and evaluated across different system settings [51, 76, 77].

The SIC has the ingredients of a map for costing implementation strategies. As a companion to the SIC, The Cost of Implementing New Strategies (COINS) was developed as a cost mapping procedure to collect implementation resource information and to assist in disentangling implementation from intervention costs. Since staff spend a considerable number of hours involved in implementation activities, the COINs was developed to calculate the fees, expenses, and person hours necessary to complete each stage [78]. Its application will identify cost and resources differences between implementation strategies for the proposed cost analyses in Aim 3 [79, 80].

For the SITT-MAT study, the SIC and COINS will be customized using a standardized adaptation approach [72]. Each of the five possible paths of implementation strategies will be operationalized. This will allow for a comparison of implementation strategy approaches and costs across programs and possible paths. If a program stops implementation strategy support at any step, their SIC measurement will discontinue. If the next step implementation strategy is initiated, a new SIC will be used to monitor the programs’ associated efforts and costs. Online data collection systems will be created to continuously collect information about the activities and costs necessary to complete the SIC and COINS.

### Data analysis

#### Specific aim 1

The analysis for Aim 1 will compare the randomized NIATx-IF versus NIATx-EF on our primary implementation outcome of the proportion of MOUD patients receiving medication within 72 h of OUD diagnosis over time. For the changes over time in the proportion of patients initiating MOUD within 72 h of diagnosis, we expect 48 new patients with OUD each quarter for each of the 54 expected sites that are randomized to either IF or EF. We will use 2-level latent growth models of 10 quarters of outcome data with individual-level binary outcomes of MOUD within 72 h. Specifically, we assume that the number of patients who start MOUD at the *i*th site and *t*th time point, *i* = 1,…,54 and *t* = 1,…,10, are all independent binomials with success probability pit on 48 individual patient trials. Here logit (pit) = ai + bi t + εit with random intercepts ai and random slopes bi = β0 + β1 Fi + εi and Fi is the indicator of facilitator condition and the effect is tested through coefficient β1.

To account for multiple comparisons in secondary analyses, we will use a false discovery rate to reflect how many subscales are assessed for each measure. In addition, we will use exploratory graphical measures, such as empirical *q*-*q* plots, to compare all the subscales’ differences by intervention condition [81]. For the analyses of the stagewise implementation to target outcomes, we will assess the degree to which all the sites eventually achieve the criterion. This criterion is defined as an IMAT Total Score ≥ 3, the presence of an integrated prescriber for MAT, and at least 50% of patients with OUD on MOUD. This is accounted for by computing the proportion of the study sites that achieve criterion in their respective phase and computing two different proportions, one for each facilitation condition, much the same way that SMART designs are evaluated for overall optimal impact.

#### Specific aim 2

We will conduct mediation and moderation analyses in the randomized trial component. For the moderation analysis, we will assess whether the size of the patient population (i.e., bed size or annual admissions) moderates the impact by testing the interaction term of size by implementation strategy. A core mediator of access to MOUD within 72-h of OUD diagnosis is the Phase 3 IMAT score which will be used to assess whether and how rapidly implementation occurs. For moderation, we will include Phase 3 IMAT score as an intermediary outcome and assess the indirect effect of the implementation strategy condition affecting the delivery of services through Phase 3 IMAT score on the MOUD rate. We will use the “product of coefficients” method for analyzing impact with a Poisson model for the MOUD rate outcome as we have shown this method is superior to the “difference of coefficients” [82]. Contextual determinant measures (CDI, ICS, ILS) and the organizations’ characteristics will also be examined for mediation and moderation of implementation outcomes and in response to specific strategies.

#### Specific aim 3

We will estimate the costs for each of the five implementation strategy paths. We will focus on obtaining a complete picture of relevant variable costs for the implementation strategies based on staff time and other variable cost such as supplies, staff travel, related contracts, ongoing IT costs, and staff space [83]. Using the activities, we will estimate labor costs using wage rates from the US Bureau of Labor Statistics that allow us to estimate costs for the local site, as well as a national average. Both are useful for replication. Local costs are informative to Washington State, while the national average is helpful for other systems or organizations interested in replicating this effort.

The costs will reflect the amount spent on implementation activities per time period. There is no empirical evidence on the optimal period of time, but there is a tension because narrow time periods require more data collection. While wider time periods require less data collection efforts, they yield cost estimates that are less precise (and perhaps less accurate). We will use quarters (90 days) as the time period. We will track implementation activities for the major organizational actors and external team members (e.g., EFs) using the COINS. We will estimate costs for the five implementation strategy paths and the stages of implementation, as measured by the SIC. Our method will allow us to document effort as well as the time it takes to move through the stages of implementation.

We will then use multivariate regression models to analyze effort and costs; although these outcomes are linked, often organizations want to understand the effect on each. For the cost analyses, we will consider different models and choose the best fitting model based upon the modified Park test, Box-Cox regression, and Hosmer and Lemeshow tests [84]. We will then link the cost per strategy to the outcomes measured in specific aim 1 (i.e., “implementation cost-effectiveness” not a cost-effectiveness analysis.

### Power calculations

For power analyses, we used Monte Carlo methods. Starting at randomization with both conditions at 20%, we have 80% power to detect a change where 54% of the patients in sites in the better facilitation condition are provided MOUD by the last quarter, compared to 45% of the patients in the poorer performing arm. This power calculation allows for moderate variation among the sites’ rates at baseline, in the slopes, and at each measurement time. The approach to the effectiveness measure, 6-month retention rate, will proceed similarly.

We will utilize latent linear growth modeling to understand the impact on the secondary composite continuous measure outcomes (e.g., IMAT; SIC). The impact will be identified as the difference in slopes for the two implementation strategy conditions, fit using maximum marginal likelihood, and tested using a Wald-type test on the difference in slopes divided by the corresponding standard error. Monte Carlo simulation was used to examine how the model’s parameters affect statistical power. With type I error of 5%, and conservative measurement error of 20% at each time point, we have 80% power to detect an effect size of 0.77 (54 randomized sites) and conservatively 0.81 (49 randomized sites). We note that in a previous head-to-head randomized implementation trial of 51 sites, we were able to find significant differences in implementation outcomes.

## Discussion

We considered but rejected two alternative designs for this multi-level stagewise implementation strategy trial. First, we discussed a design that contrasted two packaged multi-component implementation strategies tested head-to-head throughout this study for all sites, much like we conducted in an implementation trial of 51 counties in two states [51]. Unlike this two-state study, it would be inefficient in the current trial to force organizations that did not need a full-blown strategy to receive one. Second, we discussed a SMART design that used multiple randomizations for sites not meeting criteria rather than the single one used in this trial. While such a design would in theory allow more insights, multiple randomizations would sacrifice the expected large sample size of 58 sites that powerfully compare NIATx-IF versus NIATx-EF in this trial. We concluded that a study of a stepped and stagewise approach to implementation, within an innovative adaptive implementation design, although unprecedented, has the risk-reward potential for higher relevance and impact.

We also wrestled with a variety of imperfect approaches to evaluating outcomes, both as “measurement-based implementation targets” and as primary outcomes. We selected *reach* because it is standard in MOUD research and practice. The proportion of 50% on MOUD is based on the current state (~ 15%), expert recommendations, options for patient preference, and benchmarks for system-level management of chronic conditions [85]. Since 50% may prove to be a minimum threshold, we may, as a contingency, adjust this proportion or instead examine reach as a continuous variable. The criterion for *adoption* values the integrated prescriber over linked or coordinated access. This increases the likelihood that patients will not fall through the cracks between provider locations. Third, for *implementation* quality, we decided on an IMAT Total Score of 3 or more based on previous systems-level research with other organizational measures, and our recent work with the IMAT. A score of 3 represents an adequate capability. Higher scores, up to 5, reflect enhanced practice, and as a continuous measure, we expect to see IMAT scores rise through the *maintenance* phase. As a proxy for *effectiveness*, the Addiction Care Cascade provides metrics for reach, *timely access* (< 72 h to start medication), and *retention* (continuous care for 6 months). New data on 6-month retention outcomes reveal this as a bare minimum for MOUD quality care [86]. For this study, the 6-month retention rate is feasible and remains a quality standard for MOUD services research.

Efforts to combat the US opioid epidemic have focused on expanding access to MOUD. While there are indications of improved reach and adoption, an ironic gap persists—only about one third of specialty addiction treatment organizations offer MOUD. The SITT-MAT study not only advances the science of implementation but will advance our empirical understanding of how to best respond to a substance-related epidemic by checking all the boxes of expert recommendations to specify, tailor, and track implementation strategies and provides generalizable knowledge useful for systems and organizations. Our protocol leverages the enhanced rigor of advanced implementation scientific approaches to the challenge of installing and sustaining MOUD in a real-world setting, specialty addiction programs. To advance implementation research, we not only open the “black box” of implementation strategies, but we also thoroughly study the contents using a highly unique approach. This study will deploy an adaptive implementation strategy design that has never been done before. The design embeds an evaluation of non-randomized steps as well as a randomized step of implementation strategies into the overall analysis. The approach has elements of both a sequential multiple assignment randomized trial (SMART) design [49, 50, 87–89] and a criterion-based design that employs a measurement-based stepped implementation-to-target approach within an adaptive trial design to improve access to MOUD. The findings have the potential to advance drug abuse treatment research by identifying an optimization of strategies to implement MOUD.

## Supplementary Information

**Additional file 1**. The Standards for Reporting Implementation Studies (StaRI) reporting standards and checklist.

**Additional file 2. **Integrating Medications for Addiction Treatment Index.

## Data Availability

Not applicable.

## Abbreviations

CFIR: Consolidated Framework for Implementation Research
COINS: Costs of Implementing New Strategies
DDCAT: Dual Diagnosis Capability in Addiction Treatment
EF: External facilitation
EMF: Enhanced monitoring and feedback
EPIS: Exploration, Preparation, Implementation and Sustainment
HCA: Health Care Authority
HEAL: Helping to end addiction long term
ICS: Implementation Climate Scale
IF: Internal facilitation
ILS: Implementation Leadership Scale
IMAT: Integrating Medications for Addiction Treatment
IMAT-SC: Integrating Medications for Addiction Treatment in Specialty Care
MAT: Medications for Addiction Treatment
MOUD: Medications for opioid use disorder
NIATx: Network for the Improvement of Addiction Treatment
NIDA: National Institute on Drug Abuse
OUD: Opioid use disorder
RE-AIM: Reach, Effectiveness, Adoption, Implementation and Maintenance
SIC: Stages of Implementation Completion
SITT-MAT: Stagewise Implementation-To-Target–Medications for Addiction Treatment
SMSS: Sustainment Measurement System Scales
