# Supplementary material for: Stepped implementation-to-target: a study protocol of an adaptive trial to expand access to addiction medications
Source: Implement Sci. 2022 Sep 29;17:64. doi: 10.1186/s13012-022-01239-y (PMC9524103; doi:10.1186/s13012-022-01239-y)
Supplement: Supplementary file 2 — Additional file 2. Integrating Medications for Addiction Treatment Index. [file 13012_2022_1239_MOESM2_ESM.pdf]

INTEGRATING MEDICATIONS FOR ADDICTION TREATMENT IN SPECIALTY CARE (IMAT-SC)

Opioid Use Disorder Version

An Index of Capability at the Organizational/Clinic Level

YOUR PROGRAM AND AGENCY CHARACTERISTICS

DATE OF COMPLETION: _____________

NAME OF AGENCY: ____

NAME OF PROGRAM SITE: ____: LEVEL OF CARE (ASAM): _____________________________

NUMBER OF PROGRAMS WITHIN AGENCY: __________________;

ADDRESS: _____________________________________; CITY: ____________________; STATE: _____; COUNTY: _________________

KEY CONTACT

NAME: _________________________________

JOB TITLE: _________________________________

EMAIL: _________________________________

PHONE/TEXT: _________________________________

Current Capacity and Services for Medications for OUD (*In this program only & as of this date*)

# of x-waivered prescribers: _____

# of x-waivered prescribers with active patients on medication for OUD: ____

Total # of patients currently prescribed medication for OUD: ______

PROGRAM INFORMATION (PERTAINING TO THIS PROGRAM LOCATION ONLY & AS OF THIS DATE)

OF PHYSICIANS (NOT FTE BUT INDIVIDUALS ON SITE AND PERFORMING ANY CLINICAL SERVICES): ____

OF CERTIFIED NURSE PRACTITIONERS: ____

OF PHYSICIAN ASSISTANTS: ____

OF PHYSICIANS WITH ADDICTION MEDICINE BOARD CERTIFICATION (ASAM AND/OR ABPM): ____

OF PHYSICIAN PSYCHIATRISTS: ____; # OF PSYCHIATRISTS WITH BOARD CERTIFICATION IN ADDICTION (ABPM): _____

OF BEHAVIORAL HEALTH CLINICIANS WITH MENTAL HEALTH AND ADDICTION CERTIFICATION/LICENSURE: _____

BEST ESTIMATE OF PROGRAM PATIENT VOLUME AND PATIENT FUNDING TYPE (PERTAINING TO THIS PROGRAM LOCATION ONLY & AS OF THIS DATE)

IF INPATIENT/DETOX/RESIDENTIAL, # OF BEDS: _________; IF OUTPATIENT/IOP/PARTIAL HOSPITAL, AVERAGE # PATIENTS PER DAY: _______

APPROXIMATE % BY INSURANCE TYPE: MEDICAID: _____; MEDICARE: ______; PRIVATE: _______; UNINSURED: _______; OTHER: ________

IMAT-SC INDEX *version OUD 1.2*

BENCHMARK RATING SCALE: NI=Not Integrated; PI=Partially Integrated; FI: Fully Integrated

Instructions to complete the IMAT-SC Index*: Review each benchmark with team members. Based on consensus, place a checkmark in the box that best fits your current state of practice in your program site. The ratings range from: "1-Not integrated or not present" (NI) to "3- partially integrated or somewhat present but variable" (PI) to "5-fully integrated, routine and systematic" (FI). Intermediate ratings of "2" and "4", are meant for "in between" circumstances. The selection of either a "2" or a "4" should be made when the respective next level anchors of "3" or "5" are not fully met. Remember that the IMAT-SC is a quality improvement aid, not a performance exam. A candid appraisal of your existing practice will help guide your team toward any goals you wish to achieve. Giving your program conservative scores at baseline will enable your team to identify areas for quality improvement that are meaningful and achievable. The IMAT-SC is an organizational measure of capability of addiction medication treatment in behavioral health programs—with either addiction or mental health specialties. It is understood that not every item will be realistic or within your control to impact or change, or perhaps not even your program's practice goal. Nevertheless, please try to rate each item as candidly as possible and based on your team's perceptions about the current state. The estimated time to complete the IMAT-SC is 60-75 minutes.* ***THANK YOU.***

DIMENSION 1 (D1): INFRASTRUCTURE						
		**1**<br>**NI**	**2**	**3**<br>**PI**	**4**	**5**<br>**FI**
**1**	**Senior agency and program leadership, including CEO, CMO, board and clinical directors strongly support providers prescribing medications for OUD in the program**	No overt strong leadership support demonstrated at either the agency or program level		Strong program level leadership support for prescribing medications for OUD but not from senior agency leadership		Strong and overt leader support for prescribing medications for OUD at the agency and program levels
**2**	**Medical record and releases of information are privacy compliant with 42CFR and HIPAA regulations**	Our program has either not resolved or does not full understand 42CFR and HIPAA regulations		Our program has developed some workarounds to address HIPAA regulations		Our program has clear policies to access, exchange and release patient information within 42CFR and HIPAA compliance
**3**	**Insurers cover medical consultations and visits for medication management of OUD or medical services are covered by bundled contractual rates**	No provider services are covered by any insurance		Some provider services are covered, or all provider services are covered by some insurers		All reasonable provider services are covered for insured patients
**4**	**Insurers cover medications for OUD (buprenorphine and naltrexone IM) or medications are covered by bundled contractual rates**	No OUD medications are covered by any insurance		One OUD medication is covered, or both OUD medications are covered by some insurers		Both OUD medications are covered for insured patients
**5**	**Insurers cover behavioral health (addiction and/or mental health) services**	No behavioral health services are covered by any insurance		Some behavioral health services are covered, or all behavioral health services are covered by some insurers		All behavioral health services are covered for insured patients

DIMENSION 2 (D2): CULTURE AND ENVIRONMENT						
		1<br>NI	2	3<br>PI	4	5<br>FI
1	**All program staff accept and welcome equally persons on medications for OUD—no evidence for stigma or discrimination**	Most program staff, both clinical and non-clinical, negatively perceive persons on OUD meds and are reluctant to accept and welcome them		There is variation in program staff members' acceptance and empathy for persons on OUD meds but overall there is acceptance and welcome		Program-wide, there is broad-based acceptance and welcome of patients on OUD medications and for providing services to them
2	**Open display and distribution of patient informational materials about OUD and medications for OUD in common areas, therapy rooms and staff offices**	No medication for OUD informational materials for patients are visible in common spaces, therapy rooms or offices		Medication for OUD informational materials exist and are distributed to patients and family members as needed		Medication for OUD informational materials are visible in common areas, therapy rooms and offices; and routinely distributed
3	**Patients and services are visibly integrated in general program spaces and in routine operations**	Patients receiving medications for OUD are not permitted in the program		Patients receiving medications for OUD obtain these services at special days and times where patients without OUD are not scheduled, or in a location separate from the regular program		Patients receiving medications for OUD are scheduled and receive services at times concurrent with the program and in spaces available in the program
4	**All program staff believe offering medications for OUD to patients in this setting is appropriate**	Most program staff believe that offering medications for OUD in this program is inappropriate		There is variability among staff in their beliefs about the appropriateness of offering medications for OUD in this program		Program-wide, there is broad staff consensus that offering medications for OUD is appropriate in this program

DIMENSION 3 (D3): PATIENT IDENTIFICATION AND INITIATING CARE						
		1<br>NI	2	3<br>PI	4	5<br>FI
1	**All new and existing patients are screened using a standardized universal measure for opioid use risk**	No standardized measure or set of questions is used		A list of set questions about substance use issues is routinely used		A standardized and validated universal screen (e.g. TAPS, NIDA Quick Screen, DAST) is used with all new patients
2	**All patients who screen positive receive a standardized indicated assessment and if positive an OUD diagnosis is made and documented**	No standardized measure is used, and documented OUD diagnosis is variably documented		No formal standardized measure is used but OUD diagnosis is routinely documented		A standardized indicated screen (e.g. DSM5 checklist) is used to support documentation of an OUD diagnosis

DIMENSION 3 (D3): PATIENT IDENTIFICATION AND INITIATING CARE (continued)						
		1<br>NI	2	3<br>PI	4	5<br>FI
3	**All patients in the program who are on dosages of >90 mg of morphine equivalents (MMEs) for >3 months to manage chronic non-cancer pain, are reviewed and evaluated for potential OUD diagnosis and appropriateness for buprenorphine**	No review or audit of patients on >90 MMEs occurs in our practice		Review of >90 MME doses is performed by individual providers and medications for OUD offered in some cases		There is a policy for pain management that addresses MME dosing; it articulates options for tapering and alternative treatments that include medications for OUD
4	**A protocol for identification, diagnosis and treatment initiation exists for conditions commonly comorbid with OUD including other *substance use disorders***	No protocol exists for addressing other substance use disorders (e.g. alcohol, stimulants, cannabis, benzodiazepines)		Patients with OUD are assessed for other substance use problems upon initiating care and/or throughout the course of treatment		A protocol exists for screening, diagnosis, treatment planning and monitoring for other substance use disorders in addition to OUD
5	**A protocol for identification, diagnosis and treatment initiation exists for conditions commonly comorbid with OUD including *other psychiatric disorders* such as depression, anxiety, PTSD or other mental health problems**	No protocol exists for addressing other psychiatric disorders (e.g. depression, anxiety, PTSD, bipolar disorder)		Patients with OUD and in MAT are usually assessed for other mental health problems upon initiating care and/or throughout the course of treatment		A standardized protocol exists for screening, diagnosis, treatment planning and monitoring for other psychiatric disorders in addition to OUD
6	**A protocol for identification, diagnosis and treatment initiation exists for infectious diseases (ID) commonly comorbid with OUD including *HIV and HCV***	No protocol exists for addressing risk of presence of ID (e.g. HIV, HCV)		Patients with OUD and in MAT are usually screened for ID upon initiating care and/or throughout the course of treatment		A standardized protocol exists for screening, risk assessment & patient education, diagnosis, treatment planning and monitoring for ID
7	**For patients diagnosed with OUD, the prescription drug monitoring program (PDMP) is queried**	PDMP is not routinely queried		PDMP is queried but variably		PDMP is queried on all new OUD cases before initiating medications for OUD
8	**For patients diagnosed with OUD, a point-of-care toxicology test is performed, i.e. urine drug screen (UDS), with built-in and/or rapid on-site immunoassay testing**	Toxicology tests are not routinely performed		Toxicology test are inconsistently performed		Point-of-care toxicology tests are consistently performed with all new OUD cases before initiating medications for OUD

DIMENSION 3 (D3): PATIENT IDENTIFICATION AND INITIATING CARE (continued)						
		**1**<br>**NI**	**2**	**3**<br>**PI**	**4**	**5**<br>**FI**
**9**	**Patients with OUD are presented with clear treatment options, patient preferences are discussed, and a shared decision-making approach used**	Patients with OUD have no options for medications for OUD within the program		Patients with OUD have 2 options (1 medication or no medication), and these are carefully reviewed		Patients with OUD have options for 2 medications within the program and other medications outside (methadone) or no medication. The pros, cons and preferences are reviewed, and collaborative care plan chosen
**10**	**Criteria for offering medications for OUD in the program are clear; they are documented in policy, patient information sheets/brochures and consent forms; and they are highly inclusive**	Patients with OUD have no options for medications for OUD within the program		Criteria for offering medications for OUD are individual provider driven and exclude patients with other substance use, history of diversion or non-adherence or other perceived risks		Criteria for offering medications for OUD are documented and transparent; criteria focus on initiating care to reduce risk of overdose death and engage patients in care
**11**	**Three components are performed on all patients starting medications for OUD: Withdrawal symptoms are evaluated, side effects are discussed, and comfort medications to treat opioid withdrawal are made available**	None of the 3 components (withdrawal symptom evaluation, medications for OUD side effect review, comfort medications offered) are performed		2 of the 3 components (withdrawal symptom evaluation, medications for OUD side effect review, medications to treat opioid withdrawal offered) are routinely performed		All 3 components are performed (withdrawal symptom evaluation, medications for OUD side effect review, medications to treat opioid withdrawal offered) by protocol and include standardized measures and procedures (e.g. COWS; SOWs; patient informational materials; standard withdrawal medications)
**12**	**Patients choosing medications for OUD, either buprenorphine or naltrexone long acting injection, can be started on medication within 72 hours**	No patients typically are started on medications for OUD within 72 hours		The care process and review of all clinical information may take longer for some patients; Most are started within 72 hours		Protocol to initiate medications for OUD at first visit is in place

DIMENSION 3 (D3): PATIENT IDENTIFICATION AND INITIATING CARE (continued)						
		1<br>NI	2	3<br>PI	4	5<br>FI
13	**Using a protocol clear to both staff and patients, eligible patients can start the medication either at home or at the program**	No provision exists for patients to start medications for OUD at home or at the program-patients are started elsewhere and referred to us once stabilized		Protocol exists for starting medication in-office only		Protocol exists for starting medication either in-home or in-office and the approach is clear to staff and transparent to patients

DIMENSION 4 (D4): CARE DELIVERY AND TREATMENT RESPONSE MONITORING						
		1<br>NI	2	3<br>PI	4	5<br>FI
1	**Patients started on medications for OUD have 1 follow-up visit within 14 days (2 weeks)**	Follow-up visit after patients are started on medications for OUD are individually determined		Some patients are scheduled and/or attend first follow-up visit beyond 2 weeks; But most make this visit within 2 weeks		All patients are scheduled for at least 1 follow-up visit after starting medications for OUD; Those who do not attend receive outreach
2	**Patients started on MAT have at least 2 follow-up visits within 30 days (1 month)**	Follow-up visits after patients are started on medications for OUD are individually determined		Some patients are scheduled or attend 2 follow-up visits beyond 1 month; But most make these visits within the 1st month		All patients are scheduled for at least 4 follow-up visits after starting MAT; Those who do not attend receive outreach
3	**Ongoing toxicology testing, i.e. urine drug screen (UDS), is performed at least monthly, at random, and observed**	Toxicology testing is not performed once patients have started medications for OUD		Toxicology testing is performed at least monthly, but not random or observed		Toxicology testing is performed at least monthly and at random; procedures for direct observation exist
4	**The prescription drug monitoring program (PDMP) is queried at least bi-monthly**	PDMP is not queried once patients have started medications for OUD		PDMP is queried at least bi-monthly at time of visit in many but not all cases		PDMP is queried at least bi-monthly at time of visit in all cases by protocol
5	**A protocol exists for random pill or film counts for patients prescribed buprenorphine**	Pill or film counts do not occur once patients are prescribed buprenorphine		Medication counts occur variably or "for cause" once patients are prescribed buprenorphine		A protocol exists for random pill or film counts on all patients

DIMENSION 4 (D4): CARE DELIVERY AND TREATMENT RESPONSE MONITORING (continued)						
		1<br>NI	2	3<br>PI	4	5<br>FI
6	**A protocol exists, based on treatment response—including toxicology results and patient report of functioning--to adjust dose, frequency of visits and toxicological monitoring**	There is no firm protocol to adjust medications for OUD based on response		Clinical judgment is used to adjust dose, frequency of visits, and toxicology testing approach		A systematic and protocol-driven approach (e.g. OBOT Stability Index) is used to adjust dose, frequency of visits and toxicology testing approach.
7	**A systematic approach (e.g. ASAM criteria) is used to assess patient functioning and social determinants; this supports treatment planning which may include additional physical or behavioral health services either within or at another location**	No specific approach is used to evaluate patient functioning and social risk factors; no specific approach is used to guide linkage to additional services		Clinical judgment is used to evaluate patient functioning and social risk factors, and to guide linkage to additional services		A systematic and protocol-driven approach (e.g. ASAM criteria) is used to evaluate patient functioning and social risk factors, and to guide linkage to additional services
8	**A systematic approach, such as the ASAM criteria or Treatment Needs Questionnaire, is used to determine need for a more intensive level of care (residential, hospital) or setting (methadone clinic).**	No specific approach is used to determine need for a more intensive level of care or setting		Clinical judgment is used to determine need for a more intensive level of care or setting		A systematic and protocol-driven approach (e.g. ASAM or Treatment Needs Questionnaire) is used to determine need for a more intensive level of care or setting
9	**Patients are neither encouraged nor required to taper or discontinue OUD medications after a certain period of time or once stabilized or with improved functioning**	Once patients are detoxified from opioids and stable on medications for OUD we initiate the process of tapering		Patients who are stable on medications for OUD for at least 6 months and who are functioning well are encouraged to consider tapering from medication		Medications for OUD are used as a stabilization and maintenance approach; patients continue on medications with positive response, including stable and improving functioning.
10	**Six-month retention rates of patients on medications for OUD are tracked to examine our program's processes**	No retention data are tracked		Informally the program examines retention and attrition rates and refines clinical processes based on perceived trends		Six-month retention rates are routinely gathered and used to refine clinical protocols and processes

DIMENSION 5 (D5): CARE COORDINATION						
		1<br>NI	2	3<br>PI	4	5<br>FI
1	**The program uses a team based care approach to manage patients treated with medications for OUD; team members may include physicians, nurse practitioners, physician assistants, nurses, behavioral health clinicians or counselors, peer specialists, or pharmacists; and with clearly defined, written roles and responsibilities for each member of the team**	No elements of a team based care approach; the prescriber delivers most aspects of treatment using medications for OUD with some nursing support		Some elements of a team based care approach with prescribers and clinical staff working collaboratively; meetings, huddles, role specific workflows		Many elements of a team based care approach with an egalitarian model, individuals working to top of scope, and cohesive collaboration on patient care; meetings, huddles, role specific workflows; and written roles and responsibilities for each team member
2	**A registry of patients on medications for OUD is used to track patient attendance, visit planning and treatment response**	No registry of patients on medication for OUD		Some aspects of tracking panel of patients on medication for OUD are in place; patient list, set of tasks per visit, outreach criteria; not integrated in electronic health record or population health dashboard		Registry of patients on OUD medications used to systematically track patient attendance, visit planning and measuring treatment response; integrated with electronic health record and population health dashboard
3	**With the most common health care and social service partners, the program has memoranda of understanding, agreements or clear understanding of methods to coordinate care, accept referrals, refer or link patients with primary care and/or specialists (e.g. addiction, psychiatry, OB/GYN) or services (e.g. DCFS, probation and parole)**	No formal relationships with other health and social service agencies commonly involved with OUD patients in the program's medication for OUD practice		Some formal and some informal relationships with other health and social service agencies commonly involved with OUD patients in the program's medication for OUD practice		Well-coordinated network of agreements, shared documentation, and practical definitions for referral appropriateness and care coordination
4	**The program has a 42CFR and HIPAA compliant set of forms to exchange or release clinical information with patient consent**	Program focuses on 42CFR compliance only		Program has policy to manage HIPAA information along with 42CFR rules		Program and organization has legal counsel documentation supporting clear policy on 42CFR and HIPAA regulations

DIMENSION 5 (D5): CARE COORDINATION (continued)						
		1<br>NI	2	3<br>PI	4	5<br>FI
5	**Program leadership engages in regular meetings with other organizations in the geographic region to troubleshoot, improve communication and strengthen the network of care for patients on OUD medications**	No regular meetings with community coalition or other health and social service agencies commonly involved with OUD patients in the program's medications for OUD practice		Some formal and informal meetings with other health and social service agencies commonly involved with patients in the medications for OUD program; Some sense of shared mission across patients and organizational boundaries		Well-coordinated network organizations represented by leadership and key frontline personnel, with shared mission of improving communication and strengthening the network in the community

DIMENSION 6 (D6): WORKFORCE						
		1<br>NI	2	3<br>PI	4	5<br>FI
1	**X-waivered prescriber(s) onsite to prescribe medications for OUD**	No x-waivered prescribers on site		X-waivered prescribers on site and prescribing to a few patients in total (<10)		X-waivered prescribers on site and prescribing to a larger number of patients (>30)
2	**Nursing or pharmacist personnel are onsite to manage medications for OUD and nursing-related needs of patients; a nurse or pharmacist care manager model is used to perform activities during patient visits either in individual or group formats; there is coordination of care with other health care providers; patient and family education**	No nursing or pharmacist personnel involved in medications for OUD program		Nursing and/or pharmacist personnel perform some activities during patient visits but are not key team members		Nurse or pharmacist care manager model—nurse or pharmacist conduct visits, coordinate care in and outside the program, manage registry, educate patients and families, and are key team members
3	**Licensed behavioral health clinician(s) with credentials in both mental health AND addiction assessment and treatment are onsite; Have expertise to conduct evaluations, individual, group and family/couples therapies; there is expertise in integrated OUD medication for addiction treatment; Either individual clinicians have expertise in BOTH mental health AND addiction or two or more clinicians have combined expertise as a team**	No onsite behavioral health clinician involved in the medications for OUD program		Onsite behavioral health clinician(s) perform some activities but are not key team members; expert in mental health OR addiction assessment and treatment but not both; some expertise in medications for OUD		Integrated behavioral health clinician with expertise in medications for OUD; expertise in both mental health AND addiction assessment and treatment approaches, and evidence-based understanding of medications for OUD

DIMENSION 6 (D6): WORKFORCE (continued)						
		1<br>NI	2	3<br>PI	4	5<br>FI
4	**Staff or volunteer affiliation with peer recovery support group network (e.g. NA, AA, MA, Al-Anon) to educate and connect patients on medications for OUD and their support persons to these resources**	No connections with peer recovery support groups in the community		Informal efforts by some program staff to link patients on medication for OUD with peer recovery support groups in the community; some interventions focused on locating and preparing patients for meetings		Purposeful effort, including by key clinical staff or volunteers in recovery, to connect patients and their support persons to, and affiliation with peer recovery support groups in the community
5	**Administrative support to manage registry, coordination of care, liaison with other agencies, and funders**	No non-clinical administrative support for medications for OUD program		Administrative non-clinical support for financial activities including billing, budget monitoring and grant management		Administrative non-clinical support for financial activities plus patient registry maintenance, coordination of care, and liaison with other agencies

DIMENSION 7 (D7): STAFF TRAINING AND DEVELOPMENT						
		1<br>NI	2	3<br>PI	4	5<br>FI
1	**X-waivered providers/prescribers and other clinicians are actively involved in CME or equivalent continuing education and other advanced learning opportunities focused on medications for OUD, addiction and integrated behavioral health care**	X-waivered providers/prescribers and other clinicians are minimally active in advanced learning opportunities, and hide x-waiver listing from SAMHSA directory		X-waivered providers/prescribers and other clinicians are active in advanced learning opportunities, maintaining good clinical practice		X-waivered prescribers and other clinicians are active and sometimes lead advanced learning opportunities; on mission to scale up medications for OUD in their organization and field
2	**All non-clinical program staff, such as administrative and support personnel, have basic training in OUD medications**	No organized training program for non-clinical staff members on OUD medications		Optional and/or informal program to train non-clinical staff about OUD medications		Systematic and required onboarding and/or annual training program for non-clinical staff about OUD medications
3	**All staff (clinical and non-clinical) have completed training in empathy and stigma reduction for persons on medications for OUD**	No organized training program for all staff members in empathy and stigma reduction for persons on medications for OUD		Optional and/or informal training for all staff members in empathy and stigma reduction for persons on medications for OUD		Systematic and required onboarding and/or annual training program for all staff members in empathy and stigma reduction for persons on medications for OUD

IMAT-SC *OUD VERSION 1.*2: SUMMARY

PROGRAM NAME: ____; DATE COMPLETED: _____________________

D1: INFRASTRUCTURE

1.1 ____
1.2 ____
1.3 ____
1.4 ____
1.5 ____

M = ______

D2: CULTURE AND ENVIRONMENT

2.1 ____
2.2 ____
2.3 ____
2.4 ____

M = ______

D3. PATIENT IDENTIFICATION AND INITIATING CARE

3.1 ____
3.2 ____
3.3 ____
3.4 ____
3.5 ____
3.6 ____
3.7 ____
3.8 ____
3.9 ____
3.10 ____
3.11 ____

D3. PATIENT IDENTIFICATION AND INITIATING CARE (continued)

3.12 ____
3.13 ____

M = ______

D4: CARE DELIVERY AND TREATMENT RESPONSE MONITORING

4.1. ____
4.2. ____
4.3. ____
4.4. ____
4.5. ____
4.6. ____
4.7. ____
4.8. ____
4.9 ____
4.10 ____

M. = ______

D5: CARE COORDINATION

5.1 ____
5.2 ____
5.3 ____
5.4 ____
5.5 ____

M = ______

D6: WORKFORCE

6.1 ____
6.2 ____
6.3 ____
6.4 ____
6.5 ____

M = ______

D7: STAFF TRAINING AND DEVELOPMENT

7.1 ____
7.2 ____
7.3 ____

M = ______

IMAT-SC SUMMARY (n=45)

% ITEMS @NI LEVEL: _____

% ITEMS @PI LEVEL: _____

% ITEMS @FI LEVEL: _____

MEAN TOTAL IMAT-SC-SCORE:

____
